# Impact of Deep Learning-Based Reconstruction on the Accuracy and Precision of Cardiac Tissue Characterization

**DOI:** 10.3390/diagnostics16020348

**Published:** 2026-01-21

**Authors:** Margarita Gorodezky, Linda Reichardt, Tom Geisler, Marc-André Weber, Felix G. Meinel, Ann-Christin Klemenz

**Affiliations:** 1 GE HealthCare, 80807 Munich, Germany; 2 Institute of Diagnostic and Interventional Radiology, Pediatric Radiology and Neuroradiology, Rostock University Medical Center, 18057 Rostock, Germany

**Keywords:** deep learning reconstruction, cardiac mapping accuracy, cardiac tissue characterization

## Abstract

**Background/Objectives:** Interest in myocardial mapping for cardiac MRI has increased, enabling differentiation of various cardiac diseases through T1, T2, and T2* mapping. This study evaluates the impact of deep learning (DL)-based image reconstruction on the mean and standard deviation (SD) of these techniques. **Methods:** Fifty healthy adults underwent cardiac MRI, with images reconstructed using the AIR Recon DL prototype. This DL approach, which reduces noise and enhances image quality, was applied at three levels and compared to non-DL reconstructions. **Results:** Analysis focused on the septum to minimize artifacts. For T1 mapping, mean values were 988 ± 50, 981 ± 45, 982 ± 43, and 980 ± 24 ms; for T2 mapping, mean values were 53 ± 5, 54 ± 5, 54 ± 5, and 54 ± 5 ms and for T2* mapping, mean values were 37 ± 5, 37 ± 5, 37 ± 5, and 38 ± 5 ms for no DL and increasing DL levels, respectively. Results showed no significant differences in mean values for any mappings between non-DL and DL reconstructions. However, DL significantly reduced the SD within regions of interest for T1 mapping, enhancing image sharpness and registration accuracy. No significant SD reduction was observed for T2 and T2* mappings. **Conclusions:** These findings suggest that DL-based reconstructions improve the precision of T1 mapping without affecting mean values, supporting their clinical integration for more accurate cardiac disease diagnosis. Future studies should include patient cohorts and optimized protocols to further validate these findings.

## 1. Introduction

There has been a notable increase in interest in myocardial mapping in the field of cardiac magnetic resonance imaging (MRI) in recent years. A diverse range of cardiac diseases (e.g., acute myocarditis, cardiac amyloidosis or Fabry disease) can be distinguished based on myocardial tissue characterization using T1, T2 and T2* mapping [1,2]. Unfortunately the variation in mapping times is still scanner, sequence and parameter-dependent [1]. Given that the mapping times for the majority of clinical diseases exhibit minimal variability, a modest standard deviation in the acquisition of these mapping values is crucial for unambiguous identification [1].

Deep learning (DL)-based applications have the potential to overcome these limits. Some approaches focus on improved mapping acquisition by combining single-shot quantitative MRI techniques with deep learning reconstructions for faster scanning [3]. The application of DL-based reconstructions subsequent to image acquisition can reduce acquisition times by undersampling the k-space [4,5]. There is already evidence that neural networks can rapidly reconstruct T1 and T2 maps from undersampled cardiac magnetic resonance fingerprinting, but these techniques are currently not widely used for diagnoses [6]. Furthermore, DL reconstruction as a standalone post-processing step has the potential to significantly enhance the clinical integration of T1, T2, and T2* mapping techniques by reducing the standard deviation within a subject [7]. This approach could facilitate a more accurate assignment of results to cardiac diseases, thereby improving the precision and reliability of these techniques.

Several previous studies using DL-based reconstruction focused on the improvement of late gadolinium enhancement image quality and the influence on myocardial scar quantification [8,9] or DL-based reconstruction for noise reduction [10]. There are only very few studies combining DL reconstructions with standard, clinically used mapping sequences to reduce standard deviation while maintaining mapping values [7,11]. Nevertheless, the main focus of most studies is either the implementation of DL networks for reconstruction or the acceleration of the acquisition. Only small numbers of volunteers are used for these studies, and none of them uses different levels of DL and all three relaxation mapping techniques. An overview of the existing literature is added as Supplemental Table S1. The application of DL reconstructions on mapping data based on real-life data for noise reduction cannot be denied.

In this work, a cohort of 50 healthy volunteers was scanned with T1, T2 and T2* mapping, and the acquired data was reconstructed without and with different application levels of a deep learning-based image reconstruction. The influence of the DL-based reconstruction on the mean and standard deviation within a subject and in the cohort of T1, T2 and T2* mapping was systematically evaluated.

## 2. Materials and Methods

### 2.1. Ethical Approval and Participant Selection

This prospective, single-center cohort study was approved by the responsible institutional review board (University Medical Center Rostock, Germany). Written informed consent was obtained from all participants prior to enrollment. In total, 50 healthy adult volunteers in four age groups (≤34 years, 35–44 years, 45–54 years, and ≥55 years) were recruited with at least three individuals of each gender in each group to ensure a broad age distribution among the participants. The cohort consisted of 30 female and 20 male volunteers with a median age of 35 years (range: 21–66 years). The median weight was 70 kg with a range of 47–110 kg and a median height of 1.71 m with a range of 1.55–1.98 m. The median heart rate during the MRI examination was detected at 73 bpm and a range of 44–103 bpm.

Exclusion criteria were as follows: arterial hypertension, pathologies of the heart (coronary artery disease, cardiomyopathies, myocarditis, arrhythmias, etc.), diabetes mellitus, stroke, pulmonary hypertension, any chronic respiratory disease, pulmonary embolism, chronic kidney disease, any malignant or rheumatic disease, possible or known pregnancy, MR-incompatible implants, and claustrophobia.

### 2.2. MRI Acquisition Protocol and Reconstruction

Each volunteer underwent an MRI investigation with a duration of approximately 30 min on a 1.5T MRI system (SIGNA Artist, GE HealthCare, Waukesha, WI, USA). The MRI protocols included a single mid-ventricular slice acquired with the product sequences T1 Modified Look-Locker Imaging (MOLLI) 5b(3s)3b, multi-echo fast spin-echo (MEFSE) T2 and fast gradient echo T2* sequence. The acquisition parameters for all mappings were as follows: field-of-view (FOV) 40 × 32 cm^2^, slice thickness 8 mm, ARC acceleration factor 2 and a matrix size of 180 × 132 for T1 and 192 × 192 for T2 and T2*. Echo train lengths for T2 were 4 TE. For T1 mapping a nonrigid registration was applied to the reconstructed images on the scanner [12].

In addition to the product reconstruction, the prototype of the now commercially available AIR Recon DL [13] (GE HealthCare, Waukesha, WI, USA) was used to reconstruct the images. It uses a feed-forward deep convolutional neural network (CNN) that reduces noise and ringing artifacts resulting in increased signal-to-noise ratio (SNR) and edge sharpness of the images. The CNN contains over 4.4 million trainable parameters in over 10,000 kernels. The tool works in k-space eliminating truncation artifacts and reducing noise without resolution-degrading filters. It was trained with a supervised learning approach using pairs of near-perfect and synthesized lower-quality MRI images. Near-perfect images were high-resolution scans with minimal ringing and low noise, while conventional images were synthesized by introducing truncation artifacts, reduced resolution, and added Gaussian noise. To enable generalizability across all anatomies, a diverse set of training data with different contrasts and content was used. Data augmentation methods such as rotations, flips, intensity gradients, phase manipulations, and noise perturbations were used to increase robustness resulting in a database of several millions of image pairs. The network was trained with gradient backpropagation and the ADAM optimizer [14].

AIR Recon DL allows the user to set the denoising level between 0 and 1. Here, each of the acquisitions was reconstructed four times: Once the product reconstruction without any DL (noDL) and reconstructions with three levels of DL 0.3 DL (lowDL) 0.5 DL (medDL) and 0.75 DL (highDL). The selected levels were chosen to encompass a broad range of the reconstruction tool’s capabilities while minimizing the total number of reconstructions required.

### 2.3. Analysis

The cardiac maps were calculated with cvi42 (Circle Cardiovascular Imaging, Calgary, AB, Canada). The goal of this study is to solely investigate the effects of the DL reconstruction on the quantitative values. To achieve this, only data from the septum, which is least affected by artifacts and susceptibilities, was used. For the four reconstructions, similar regions of interest (ROIs) were drawn freehand in the septum of the myocardium. For all drawn ROIs the mean value and the standard deviations (SDs) were obtained.

To assess the variation within the reconstruction methods, two comparisons were performed:

First, the variation within the ROI of each individual was assessed by obtaining the SD of the values within the ROI of each volunteer (SDroi) directly from cvi42. This way the variance for each subject was established.

Furthermore, the mean values and their SD over the whole population of volunteers (SDpop) were calculated. The goal here was to investigate whether the range of the values would be affected by the different reconstructions.

The T1, T2 and T2* values of the three DL reconstructions were compared to the non-DL reconstruction with the Wilcoxon signed rank sum test. In cases of the DL reconstructions being significantly different from the non-DL reconstruction, an additional test was performed comparing the three DL reconstructions to each other. To account for multiple testing of six different variants, a Bonferroni-adjusted significance level of *p* < 0.008 (0.05/6) was considered to indicate statistical significance.

## 3. Results

**Figure 1:**
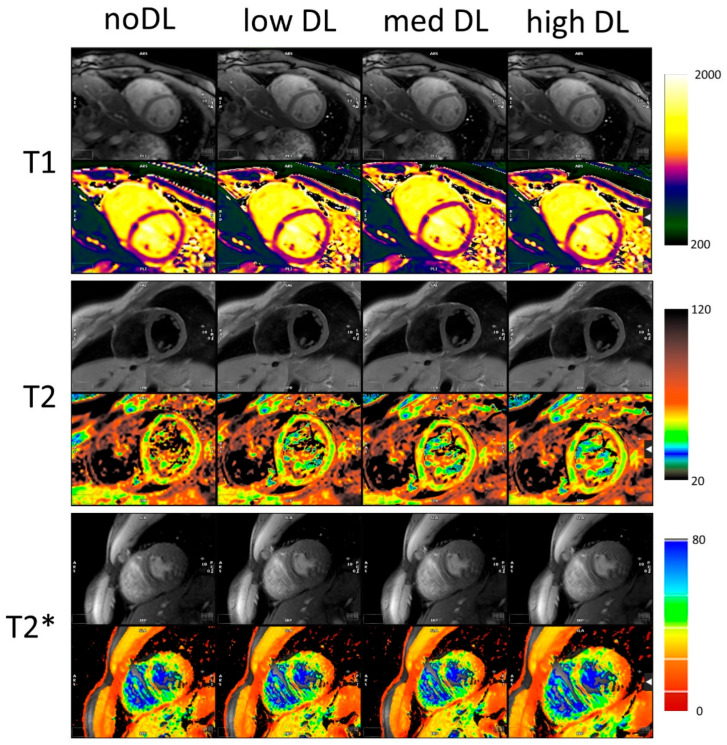
Original images and maps for outliers for T1, T2 and T2*. The DL reconstruction reduces the noise and mitigates the effect of artifacts. All colourmaps in ms.

**Figure 2:**
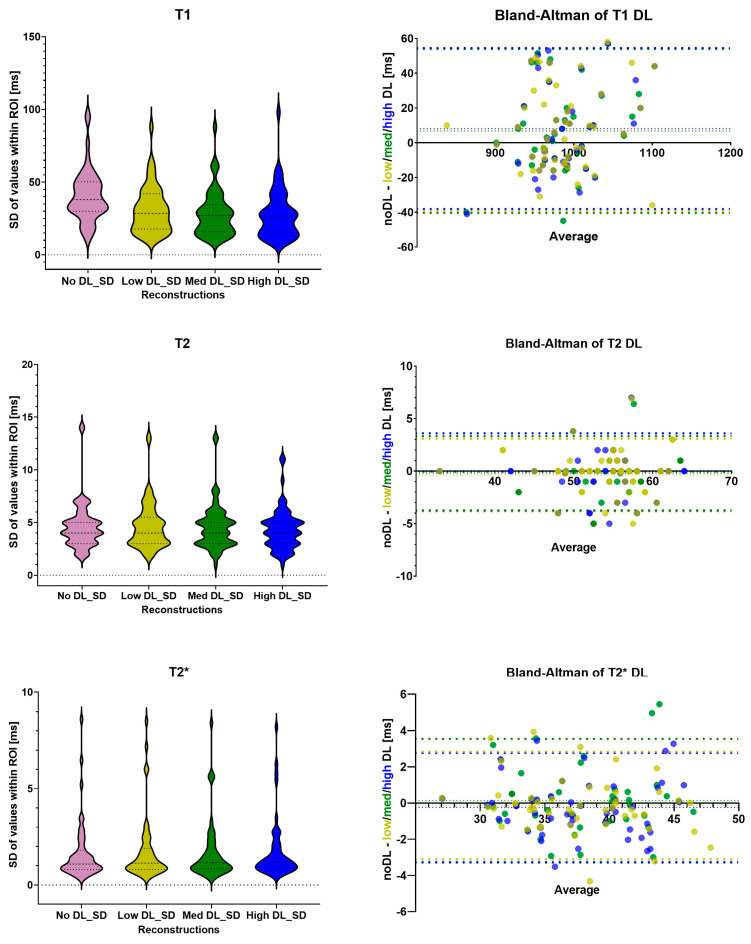
The distribution of the SDroi for T1, T2 and T2* values as violin plots; Bland–Altman plots to compare the noDL reconstruction to the three DL reconstructions. SD = standard deviation of region of interest; DL = deep learning; ROI = region of interest; low DL = shown in light green; med DL = shown in dark green; and high DL= shown in blue.

Figure 1 shows the maps and a single DICOM image per relaxation type and reconstruction. For each mapping the dataset with the biggest differences between the methods was chosen. These cases also demonstrated a lower image quality compared to the average acquisition in this study. The results of the T1, T2 and T2* statistical analysis are summarized in Table 1. All individual measured values and their SDroi are shown in Supplementary Tables S2 and S3, respectively. The Bland–Altman comparison of the noDL to the three DL reconstructions and the distribution of the SDroi are visualized in Figure 2 for T1, T2 and T2*, respectively.

### 3.1. T1 Mapping

The mean and SDpop of T1 values are 988 ± 50, 981 ± 45, 982 ± 43 and 980 ± 48 ms for noDL, lowDL, medDL and highDL, respectively.

There are no statistical differences comparing noDL to lowDL, midDL and highDL with *p* > 0.08 and a bias of 7.2, 6.8 and 8.2 ms, respectively. The higher T1 values for the noDL reconstruction might be caused by partial volume effects due to reduced sharpness of the myocardial edges affecting the motion correction.

While no statistical differences in T1 values were found comparing noDL to the three DL reconstructions, all three DL reconstructions showed a significant difference in SDroi. The median of the SDroi has significantly (*p* < 0.0001) decreased from 37 ms for noDL to 27, 25 and 24 ms for lowDL, medDL and highDL reconstructions. Within the different DL reconstructions, the interquartile range has decreased: the first quartile is reduced from 18 to 15, and the third quartile has decreased from 40 to 34 ms from lowDL to both medDL and highDL, respectively.

The T1 values of the three DL reconstructions are similar, but the distribution of the SDroi exhibits variation between the lowDL reconstruction and the other two DL reconstructions. In the next step the DL reconstructions were compared with each other. The differences between the lowDL and both midDL and highDL are significant (*p* < 0.0002) but not significant between midDL and highDL (*p* = 0.03).

### 3.2. T2 and T2* Mapping

The mean and SDpop of T2 values are 53 ± 5, 54 ± 5, 54 ± 5 and 54 ± 5 ms for noDL, lowDL, medDL and highDL, respectively. For T2 mapping, there are no significant changes observed, neither in the T2 values nor in the SDroi between noDL and the three DL reconstructions (*p* > 0.5). The bias of the T2 values is negligible being less than 0.4 ms. While the interquartile range has not changed for T2, the outliers of the SDroi are smaller for the highDL reconstruction.

The mean and SDpop of T2* values are 37 ± 5, 37 ± 5, 37 ± 5 and 38 ± 5 ms for noDL, lowDL, medDL and highDL, respectively. For T2* mapping there are no significant differences neither in the values nor in the SDroi (*p* > 0.1), while the bias is negligible being less than 0.3 ms. The interquartile ranges are similar between the reconstructions.

## 4. Discussion

For T1 and T2* the measured ranges were within the values found in the literature; for T2 they were at the higher end of the literature values, which is expected for the MEFSE technique compared to the more commonly used T2 prep sequences [15,16,17,18,19,20]. There were no significant changes to T1, T2 nor T2* values due to the AIR Recon DL reconstruction. The nonsignificant decrease in T1 values from noDL to all DL reconstructions might be caused by different image registrations leading to increased partial volume effects. The image registration is applied in image space after reconstruction, and the sharper borders between myocardium and blood pool of the DL reconstruction might result in a more accurate registration of the images. Particularly for maps with a thin myocardium where drawing an ROI can be tricky, this might avoid the blood pool of slightly misregistered images affecting the myocardium values. This effect might be especially useful in patients with dilated myocardium. The DL reconstruction can be particularly beneficial for patients with diffuse pathology where a higher SNR and a better registration might increase diagnostic confidence.

One major problem for clinical implementation of mapping data is the minimal variability of mapping values in clinical diseases [1,21]. Therefore, a minimal standard deviation is crucial for the exact identification of cardiomyopathies. The standard deviation within the ROIs was significantly smaller for the DL reconstructed images compared to noDL for T1 mapping but not for T2 and T2* mapping. While T2 and T2* have sufficient SNR and the DL reconstruction had no added value, the T1 maps’ quality benefited from the DL reconstructions. Given that there were no significant changes in SDroi between midDL and highDL, a medium DL of 0.5 seems sufficient.

High precision in cardiac T1 mapping is crucial for detecting and quantifying subtle differences in myocardial tissue properties. It reduces measurement variability, which is essential for longitudinal studies, therapy monitoring, and early disease detection. Inaccurate or imprecise measurements can lead to misclassification and undermine diagnostic confidence. While accuracy ensures that measured values reflect true tissue characteristics, clinical decision-making often depends more on precision. Poor precision, by contrast, can obscure meaningful trends. For this reason, MOLLI is widely preferred over Saturation recovery single-shot acquisition (SASHA) [22,23] in clinical practice: its superior precision and reproducibility provide greater confidence in longitudinal assessments and multi-center studies, despite SASHA offering marginally higher accuracy under certain conditions [24,25]. The reduced standard deviation within the ROIs as shown in this study demonstrates an increased precision of the measurement on a subject basis.

### Limitations and Future Work

One limitation of this study is that it is based on a single-center cohort and relies exclusively on data and a deep learning tool from a single manufacturer, which may limit the generalizability of the findings. The DL-reconstruction is proprietary and can only be performed on the same vendor. In future work, the reproducibility can be tested at a different site, scanner or field strength. Only healthy volunteers are examined, and, therefore, no statement about the improved precision in patients with cardiomyopathy can be made. However, because of the generalist nature of the DL tool, it is not expected to be affected by pathological values or physiology. In future work this study can be expanded to different patient cohorts and pathologies. The tool is expected to perform similarly in patients with abnormal mapping values. To eliminate potential susceptibility effects from the lateral wall, the analysis was restricted to the septum. Since susceptibility does not influence noise levels, the impact of the DL reconstruction is expected to remain consistent. Regions were excluded because visual assessment could be compromised by artifacts unrelated to noise. The imaging protocols were not adapted for DL use to avoid differences in values caused by protocol changes. In this initial step, this study aimed to verify that the original mapping values are not manipulated by the DL reconstruction. In further work, an optimization of mapping protocols and subsequent variations will be interesting and might have the potential to reduce standard deviations even in T2 and T2* mapping leading to either an increased spatial or temporal resolution. The protocols can be optimized to reduce the acquisition duration for segmented acquisitions of T2 and T2*, or increase the spatial or temporal resolution for the single-shot acquisition of T1. The effects of the shorter acquisition on image quality and artifact reduction can be evaluated particularly for patients struggling to hold their breath. Alternatively, increasing the resolution may enhance the ability to investigate fibrotic conditions.

Nevertheless, we believe that our work mainly contributes to making cardiac mapping more robust and therefore might help to improve clinical implementation.

## 5. Conclusions

This study demonstrated that the T1, T2 and T2* mapping values are not affected by the DL reconstruction. The standard deviation in the individual ROIs was significantly decreased for T1 but not for T2 and T2* values. Decreased SDroi might provide higher diagnostic confidence, particularly in patients with a dilated myocardium or with diffuse disorders. This study confirms that this DL reconstruction tool does not significantly affect the mapping values and provides the ground for further work of utilizing the tool to enhance mapping.

## Figures and Tables

**Table 1:** Statistical results of the comparison between noDL and the three DL reconstructions for T1, T2 and T2*. SDpop = Standard deviation of the values; SDroi = standard deviation of region of interest; and DL = deep learning.

		T1			T2			T2*		
		low DL	med DL	high DL	low DL	med DL	high DL	low DL	med DL	high DL
	Wilcoxon test *p*-value mapping values	0.08	0.17	0.06	0.05	0.39	0.69	0.16	0.84	0.13
Bland–Altman	Bias	7.2	6.8	8.2	−0.4	−0.2	−0.1	−0.1	0.1	−0.2
	SDpop of bias	23.8	24.1	23.6	1.8	1.8	1.9	1.5	1.7	1.5
	95% limits of agreement									
	From	−39.4	−40.5	−38.2	−3.8	−3.7	−3.7	−3.1	−3.3	−3.3
	To	53.8	54.0	54.5	3.1	3.3	3.6	2.8	3.5	2.8
	Wilcoxon test *p*-value SDroi	<0.0001	<0.0001	<0.0001	0.88	0.84	0.04	0.20	0.84	0.12

## Data Availability

The original contributions presented in this study are included in the article/Supplementary Materials. Further inquiries can be directed to the corresponding author.

## Supplementary Materials

The following supporting information can be downloaded at https://www.mdpi.com/article/10.3390/diagnostics16020348/s1, Table S1: Literature review summary table; Table S2: Normal ranges and values over all healthy volunteers for T1, T2 and T2*; Table S3: Standard deviation within the ROIs (SDroi)for T1, T2 and T2*.

## Abbreviations

MRI	Magnetic resonance imaging
DL	Deep learning
SNR	Signal-to-noise ratio
CNN	Convolutional neural network
TE	Echo time
SDpop	Standard deviation of values over the healthy volunteer population
ROI	Region of interest
SDroi	Standard deviation of region of interest within each volunteer
