# Supplementary material for: Impact of Deep Learning-Based Reconstruction on the Accuracy and Precision of Cardiac Tissue Characterization
Source: Diagnostics (Basel). 2026 Jan 21;16(2):348. doi: 10.3390/diagnostics16020348 (PMC12840211; doi:10.3390/diagnostics16020348)
Supplement: Supplementary file 1 [file diagnostics-16-00348-s001.zip › diagnostics-4075967-supplementary.pdf]

Supplemental Table S1: Literature summary overview

Study	Dataset	Methodology	Results	Limitations and Gap
**Accelerated cardiac T1 mapping in four heartbeats with inline MyoMapNet: a deep learning-based T1 estimation approach** (Guo et al., *Journal of Cardiovascular Magnetic Resonance* ,2022)** “”	Training: 607 patients (native + post-contrast MOLLI data); Testing: 61 patients; Prospective validation: 27 subjects; Phantom studies;	• Development of MyoMapNet, a fully connected neural network (FCNN) trained on MOLLI data to estimate myocardial T1 from only four T1-weighted images acquired after a single inversion (LL4).<br>• Inline reconstruction implemented on the scanner.<br>• Compared against MOLLI for native/post-contrast T1 and ECV at 3T	• MyoMapNet enabled accurate T1 mapping in 4 heartbeats with excellent agreement to MOLLI (small biases for myocardium and blood).<br>• Precision slightly lower but clinically acceptable.<br>• Inline reconstruction feasible, enabling substantial scan-time reduction.	• Training relied on MOLLI (inherits MOLLI bias)<br>• single-vendor, single-field strength (3 T);<br>• limited evaluation across specific cardiomyopathies; slightly reduced precision vs. MOLLI for blood T1<br>• Only T1 Mapping<br>• Focus on scan time reduction
**Evaluating Second-Generation Deep Learning Technique for Noise Reduction in Myocardial T1-Mapping Magnetic Resonance Imaging** (Sawamura et al, *Diseases*, 2025)	36 patients and phantom study with 7 homemade phantoms with different Gd-DOTA dilution ratios	• Evaluation of super-resolution deep learning-based reconstruction (SR-DLR) regarding noise reduction and measurement consistency<br>• MOLLI sequence was used as basis<br>• Quantitative analysis of T1 values, standard deviation and intraclass correlation coefficient	• No impact of SR-DLR on T1 values<br>• Significant reduction of SD	• Only T1 Mapping<br>• Single vendor, single field strength (1.5T)<br>• Relatively small cohort
**Deep Learning Reconstruction for Cardiac Magnetic Resonance Fingerprinting T1 and T2 Mapping** (Hamilton et al., *Magn Reson Med*, 2021)	Simulated dataset of ~8 million cMRF signals covering wide T1/T2 ranges and variable heart rates and *In vivo* cMRF data from 58 healthy subjects	• Fully connected neural network directly mapping undersampled cMRF signal timecourses and ECG RR intervals to voxel-wise T1 and T2 values<br>• Training uses Bloch simulations with realistic spiral undersampling artifacts (“pseudo-noise”) and random phase shifts	• Normalized root mean square error <1% for T1 and <4–6% for T2 across tissues in simulations<br>• Mean *in vivo* myocardial differences vs. dictionary matching: 3.6 ms (T1) and −0.2 ms (T2)<br>• Reconstruction time reduced from >4	• CMR fingerprinting not MOLLI sequence<br>• Trained primarily on simulated data; generalization to pathological tissue distributions not extensively validated

			minutes to ~336 ms per slice	
**Rapid High-Fidelity T2 Mapping Using Single-Shot Overlapping-Echo Acquisition and deep learning reconstruction** (Yang et al., *Magn Reson Med*, 2023)	Phantom experiments *and in vivo* human brain and body MRI data	• Single-shot sequence acquiring multiple overlapping spin echoes in one readout<br>• Model-based reconstruction disentangles overlapping echo contributions<br>• Designed to achieve rapid T2 mapping with reduced sensitivity to motion	• Produces high-fidelity T2 maps in a single shot<br>• Significantly reduced scan time compared to conventional multi-echo T2 mapping<br>• Good agreement with reference T2 values in phantom and in vivo studies	• No DL reconstruction used -> development of new acquisition sequence<br>• Only T2<br>• Primarily validated for specific anatomical targets (not for heart)
**A Self-Supervised Deep Learning Reconstruction for Shortening the Breathhold and Acquisition Window in Cardiac Magnetic Resonance Fingerprinting** (Hamilton et al., *Frontiers in Cardiovascular Medicine*, 2022)	Simulated cardiac MRF data and ISMRM/NIST phantom and 18 healthy subjects and 10 cardiomyopathy patient	• **DIP-MRF**: combines low-rank subspace modeling with a deep image prior<br>• Network trained *per scan* using only the acquired undersampled k-space data (no pretraining)<br>• Enables shortened breathhold (5 heartbeats vs. 15) and reduced acquisition window	• Improved noise and aliasing suppression in vivo<br>• Consistent myocardial T1/T2 between long and short breathhold scans (bias: −9 ms T1, +2 ms T2)	• CMR fingerprinting not MOLLI sequence<br>• Computationally expensive due to per-scan optimization<br>• Does not explicitly correct for $B1^+$ inhomogeneity
**A Myocardial T1-Mapping Framework with Recurrent and U-Net Convolutional Neural Networks** (Jeelani et al., IEEE ISBI, 2020)	Fully sampled MOLLI T1-mapping data from 45 healthy subjects (basal & mid slices, pre and post contrast)	• recurrent CNN (CRNN) reconstruction network exploiting spatial, temporal, and iterative recurrence with a data-consistency layer to suppress aliasing and noise in undersampled inversion-recovery image series;<br>• U-Net-based mapping network that performs spatially regularized, data-driven mapping from reconstructed time-series images to a single T1 map, replacing pixel-wise three-parameter nonlinear fitting	• Improved noise robustness and precision compared to pixel-wise three-parameter fitting after both k-t SLR and CRNN reconstruction; higher SSIM in myocardial region<br>• narrower Bland–Altman confidence intervals indicating higher precision	• Trained and evaluated only on **healthy subjects**<br>• retrospective undersampling (no prospective accelerated acquisition)<br>• ground truth T1 maps derived from pixel-wise fitting of fully sampled data (not an independent reference)<br>• evaluated only on MOLLI sequence and 2D slices

Sup Table S2: Normal ranges and values over all healthy volunteers for T1, T2 and T2*

Normal ranges

	T1 map				T2 map				T2* map			
	noDL	lowDL	medDL	highDL	noDL	lowDL	medDL	highDL	noDL	lowDL	medDL	highDL
**mean**	**988**	**981**	**982**	**980**	**53**	**54**	**54**	**54**	**37**	**37**	**37**	**38**
**SD**	**50**	**45**	**43**	**48**	**5**	**5**	**5**	**5**	**5**	**5**	**5**	**5**
1	994	946	941	961	64	63	64	61	36	41	39	39
2	941	930	968	972	50	55	54	52	39	40	40	40
3	1007	992	989	986	48	48	48	48	34	34	34	34
4	976	930	933	927	56	58	56	55	42	42	42	43
5	1082	1067	1071	1118	54	55	55	55	31	31	31	31
6	923	934	935	941	58	59	58	60	46	47	41	43
7	981	997	992	993	52	52	53	52	42	39	41	42
8	966	982	982	982	56	54	54	54	40	40	40	40
9	1125	1081	1081	1081	61	61	61	61	34	35	35	35
10	1001	981	983	983	57	57	56	58	42	43	44	44
11	980	928	929	933	52	52	52	52	44	44	43	43
12	989	997	998	997	53	53	53	53	34	34	32	35
13	940	953	961	956	57	57	57	57	45	43	44	44
14	964	1009	984	934	42	44	42	40	36	38	36	38
15	1072	1015	1015	1014	58	58	58	58	33	33	32	33
16	989	981	981	987	54	54	52	53	33	33	32	33
17	1097	1069	1061	1051	33	33	33	33	33	29	29	31
18	1049	1022	1021	1021	49	49	49	49	31	32	32	32
19	1095	1075	1075	1075	61	54	54	54	36	32	33	33
20	947	926	926	927	52	48	48	48	31	31	31	31
21	986	951	951	950	55	54	53	54	40	40	40	42
22	954	967	967	967	56	56	56	56	36	38	38	37
23	985	998	998	999	45	45	45	45	42	42	41	44
24	993	1019	1022	1017	57	57	56	55	33	34	34	34
25	973	972	971	951	57	56	56	56	47	49	41	43
26	1030	1020	1020	1021	56	55	55	55	40	39	40	41
27	843	883	884	833	56	55	55	56	39	38	37	38
28	992	946	946	946	55	56	58	60	45	45	45	45
29	995	982	983	983	54	57	57	57	42	44	43	43
30	960	963	965	963	56	56	56	57	37	36	36	36
31	961	965	966	966	48	48	49	48	42	45	45	44
32	967	964	965	965	57	57	57	57	34	34	36	36
33	1000	989	989	989	62	62	62	62	34	35	37	38
34	958	968	968	967	55	55	55	55	41	40	40	40
35	1025	1016	1016	1015	51	51	51	51	37	38	38	37
36	984	971	971	971	55	55	55	55	34	34	34	34
37	901	901	902	902	52	55	57	56	37	37	37	38
38	978	973	972	972	51	52	50	51	40	40	40	40
39	1008	1023	1023	1022	55	54	55	54	41	42	42	42
40	971	980	980	981	57	57	57	57	38	38	38	38
41	948	952	964	964	49	49	49	49	32	31	32	33
42	1009	1011	1011	1012	59	62	62	62	37	37	37	38
43	969	923	922	922	50	50	51	49	33	30	30	30
44	1017	1037	1037	1036	46	50	50	50	34	34	35	36
45	1002	1007	1006	1004	56	57	57	56	36	35	35	35
46	943	951	951	951	56	60	60	60	39	36	37	37
47	1031	989	988	987	54	55	54	55	27	27	27	27
48	984	999	997	995	48	48	48	48	35	36	35	36
49	1066	1062	1061	1061	56	56	56	56	41	40	40	40
50	933	925	924	924	56	58	57	58	46	46	47	45

Sup Table S3: Standard deviation within the ROIs (SDroi)for T1, T2 and T2*

	**T1 map**				**T2 map**				**T2* map**			
	**noDL**	**lowDL**	**medDL**	**highDL**	**noDL**	**lowDL**	**medDL**	**highDL**	**noDL**	**lowDL**	**medDL**	**highDL**
**mean**	**37**	**27**	**25**	**24**	**53**	**54**	**54**	**54**	**37**	**37**	**37**	**38**
**SD**	**18**	**16**	**16**	**17**	**5**	**5**	**5**	**5**	**5**	**5**	**5**	**5**
1	33	47	39	58	64	63	64	61	36	41	39	39
2	32	34	13	16	50	55	54	52	39	40	40	40
3	47	37	29	42	48	48	48	48	34	34	34	34
4	49	35	30	29	56	58	56	55	42	42	42	43
5	58	88	88	98	54	55	55	55	31	31	31	31
6	45	30	28	29	58	59	58	60	46	47	41	43
7	29	26	20	17	52	52	53	52	42	39	41	42
8	39	16	14	12	56	54	54	54	40	40	40	40
9	97	61	59	58	61	61	61	61	34	35	35	35
10	38	42	42	41	57	57	56	58	42	43	44	44
11	46	17	16	19	52	52	52	52	44	44	43	43
12	30	12	12	13	53	53	53	53	34	34	32	35
13	43	13	16	13	57	57	57	57	45	43	44	44
14	67	56	36	52	42	44	42	40	36	38	36	38
15	47	17	16	16	58	58	58	58	33	33	32	33
16	53	29	28	35	54	54	52	53	33	33	32	33
17	93	43	33	27	33	33	33	33	33	29	29	31
18	51	27	27	26	49	49	49	49	31	32	32	32
19	50	45	44	43	61	54	54	54	36	32	33	33
20	45	35	34	33	52	48	48	48	31	31	31	31
21	77	19	19	19	55	54	53	54	40	40	40	42
22	33	13	11	10	56	56	56	56	36	38	38	37
23	35	42	41	40	45	45	45	45	42	42	41	44
24	51	55	61	52	57	57	56	55	33	34	34	34
25	22	22	18	26	57	56	56	56	47	49	41	43
26	52	49	48	47	56	55	55	55	40	39	40	41
27	60	64	63	30	56	55	55	56	39	38	37	38
28	33	11	11	10	55	56	58	60	45	45	45	45

29	39	23	21	20	54	57	57	57	42	44	43	43
30	37	20	25	12	56	56	56	57	37	36	36	36
31	14	29	28	28	48	48	49	48	42	45	45	44
32	35	18	17	16	57	57	57	57	34	34	36	36
33	22	22	13	11	62	62	62	62	34	35	37	38
34	19	19	16	13	55	55	55	55	41	40	40	40
35	30	14	10	8	51	51	51	51	37	38	38	37
36	30	27	25	23	55	55	55	55	34	34	34	34
37	29	23	23	24	52	55	57	56	37	37	37	38
38	55	46	45	45	51	52	50	51	40	40	40	40
39	30	31	29	28	55	54	55	54	41	42	42	42
40	16	15	13	10	57	57	57	57	38	38	38	38
41	20	13	20	19	49	49	49	49	32	31	32	33
42	17	17	15	13	59	62	62	62	37	37	37	38
43	44	43	41	40	50	50	51	49	33	30	30	30
44	40	33	30	27	46	50	50	50	34	34	35	36
45	11	12	10	9	56	57	57	56	36	35	35	35
46	31	33	31	30	56	60	60	60	39	36	37	37
47	57	37	33	29	54	55	54	55	27	27	27	27
48	30	28	27	26	48	48	48	48	35	36	35	36
49	34	25	21	17	56	56	56	56	41	40	40	40
50	38	34	33	32	56	58	57	58	46	46	47	45
